# Sialic Acids and Their Influence on Human NK Cell Function

**DOI:** 10.3390/cells10020263

**Published:** 2021-01-29

**Authors:** Philip Rosenstock, Thomas Kaufmann

**Affiliations:** Institute for Physiological Chemistry, Martin-Luther-University Halle-Wittenberg, Hollystr. 1, D-06114 Halle/Saale, Germany; thomas.kaufmann270@gmail.com

**Keywords:** sialic acids, sialylation, NK cells, Siglecs, NCAM, CD56, sialyltransferases, NKp44, Nkp46, NKG2D

## Abstract

Sialic acids are sugars with a nine-carbon backbone, present on the surface of all cells in humans, including immune cells and their target cells, with various functions. Natural Killer (NK) cells are cells of the innate immune system, capable of killing virus-infected and tumor cells. Sialic acids can influence the interaction of NK cells with potential targets in several ways. Different NK cell receptors can bind sialic acids, leading to NK cell inhibition or activation. Moreover, NK cells have sialic acids on their surface, which can regulate receptor abundance and activity. This review is focused on how sialic acids on NK cells and their target cells are involved in NK cell function.

## 1. Introduction

### 1.1. Sialic Acids

N-Acetylneuraminic acid (Neu5Ac) is the most common sialic acid in the human organism and also the precursor for all other sialic acid derivatives. The biosynthesis of Neu5Ac begins in the cytosol with uridine diphosphate-N-acetylglucosamine (UDP-GlcNAc) as its starting component [1]. It is important to understand that sialic acid formation is strongly linked to glycolysis, since it results in the production of fructose-6-phosphate (F6P) and phosphoenolpyruvate (PEP). With the presence of F6P, the hexosamine pathway is initiated, ultimately leading to the formation of UDP-GlcNAc and glutamate from F6P and glutamine through several enzymatic steps [2]. PEP itself is an essential substrate further downstream in the pathway. 

The key enzyme of the sialic acid biosynthesis is the bifunctional UDP-N-Acetylglucosamine 2-epimerase/N-Acetylmannosamin kinase (GNE), catalyzing the first two reactions. The most expressed isoform of this enzyme is a protein with a length of 722 amino acids, which can be divided into an N-terminal epimerase domain (~AA 1–400) and a C-terminal kinase domain (~AA 401–722) [3,4]. It is known that the GNE is present in different oligomeric states (dimer and tetramer), influencing its catalytic activity [5]. The biologically-active form is the tetrameric state, which is capable of catalyzing the epimerase and the kinase reaction. The dimeric state is only able to catalyze the kinase reaction, but not the epimerase reaction [6]. An important regulatory mechanism of the GNE is the negative feedback inhibition by binding of the downstream product cytidine monophosphate(CMP)-Neu5Ac at the allosteric site [7]. Although the GNE is a bifunctional enzyme with the ability to catalyze two enzymatic reactions, the feedback inhibition is only affecting the UDP-GlcNAc epimerase activity [8]. It also seems that the product of the first reaction (UDP) remains in the catalytic site, which also increases the stability of the enzyme [6]. 

**Figure 1:**
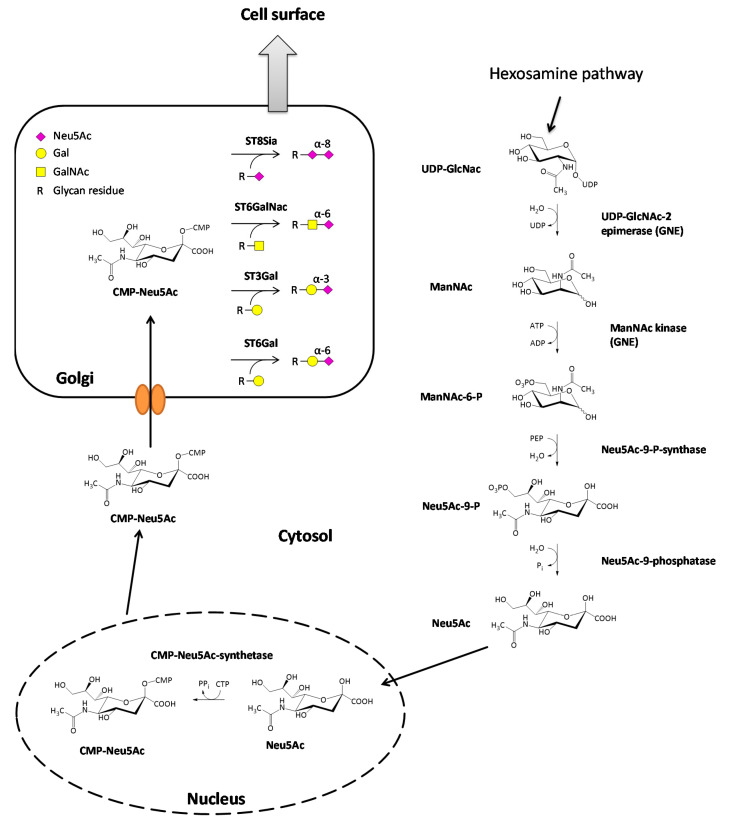
Biosynthesis of sialic acids and attachment to glycan structures. The most common sialic acid in humans is N-Acetylneuraminic acid (Neu5Ac), which is derived from uridine diphosphate-N-acetylglucosamine (UDP-GlcNAc) in the cytosol. In the nucleus, Neu5Ac reacts further with cytidine triphosphate CTP, and the activated form cytidine monophosphate(CMP)-Neu5Ac is synthesized. CMP-Neu5Ac is then transported to the Golgi apparatus where four groups of sialyltransferases (ST8Sia, ST6GalNAc, ST3Gal, ST6Gal) exist, which attach Neu5Ac to glycan residues of glycolipids or glycoproteins. Afterward, the modified molecules are transported to the cell surface.

The synthesis of sialic acids starts with the epimerization of UDP-GlcNAc, leading to the formation of N-acetylmannosamine (ManNAc) through cleavage of the UDP nucleotide unit of the molecule. Immediately afterward, a γ-phosphate group of an ATP is transferred to the ManNAc in a kinase reaction resulting in ManNAc-6-phosphate and ADP [3]. Further downstream, ManNAc-6-phosphate reacts with phosphoenolpyruvate (PEP), derived from glycolysis, in an aldol addition to Neu5Ac-9-phosphat [9]. The reaction is catalyzed by the Neu5Ac-9-phosphate-synthase and energetically driven by the release of the phosphate group of the PEP. This reaction is followed by the cleavage of the phosphate group on the C9-position of the amino sugar catalyzed by the Neu5Ac-9-phosphatase resulting in the formation of N-acetylneuraminic acid (Neu5Ac). The final step of the sialic acid synthesis is the formation of CMP-Neu5Ac in the nucleus of the cell by the CMP-Neu5Ac-synthetase [10]. Almost all mammalian species can also synthesize N-glycolylneuraminic acid (Neu5Gc) from CMP-Neu5Ac. One exception are humans because they have a loss of function mutation in the enzyme converting CMP-Neu5Ac to CMP-Neu5Gc [11]. However, Neu5Gc taken up by dietary sources can be incorporated in human cells and is accumulated in some cancer types [12]. Nevertheless, the amount of Neu5Ac is relatively low, and Neu5Ac is the most common sialic acid in humans. From the nucleus, CMP-Neu5Ac is transported to the Golgi apparatus, passing its membrane through specific carrier proteins. Once located in the lumen of the Golgi, CMP-Neu5Ac then serves as a substrate for various sialyltransferases [13]. The sialic acid biosynthesis is illustrated in Figure 1. 

Sialyltransferases themselves are a family of enzymes that catalyze sialylation, the attachment of a CMP-activated sialic acid scaffold onto glycoconjugates, through the loss of the nucleotide unit. In humans, there are 20 different sialyltransferases identified carrying out the formation of different glycosidic bonds. Due to the formation of different glycosidic bonds, the 20 sialyltransferases are separated into four different families. First of all, the family of sialyltransferases (ST8Sia; EC 2.4.99.8) catalyze the formation of an α-2,8-glycosidic bond between an already existing sialic acid and Neu5Ac. The second family of sialyltransferases (STGalNAc6; EC 2.4.99.3) transfers Neu5Ac onto GalNAc forming an α-2,6-glycosidic bond between the sialic acid and the sugar component. The last two families are composed of the β-Galactoside α-2,6-(ST6Gal, EC 2.4.99.1) and the β-Galactoside α-2,3-sialyltransferases (ST3Gal, EC 2.4.99.4), resulting in the formation of the corresponding glycosidic bond between the sialic acid (Neu5Ac) and galactose [14,15,16] (Figure 1). 

Although different sialyltransferases catalyze reactions between the same two carbohydrate substrates, they differ in their functions, since they are able to react with glycans from different substrates. ST8Sia I and ST8Sia V almost exclusively recognize glycan structures on gangliosides as substrates [17,18], whereas ST8Sia VI prefers O-glycans as substrate [19]. Two special sialyltransferases that should further be highlighted are ST8Sia II and ST8Sia IV. Their main function is the formation of the polysialic acid (polySia) modification on the neural cell adhesion molecule (NCAM) [20]. It is widely known that NCAM and especially its polysialylated form are associated with cancer and tumor progression [21]. Furthermore, it was shown that during neoplastic transformation, the activity of sialyltransferases is increased, leading to a stronger sialylation pattern on the surface of cancer cells [22,23]. Additionally, aberrant sialylation of glycans, shortening of O-glycosylproteins glycan chains, and reduced O-acetylation of sialic acids could have been shown [24,25]. It is also known that this deviating sialylation of glycan structures influences key processes during tumor progression, such as invasion and metastasis. Some sialylated glycans are useful prognostic markers of malignant disease states. Cancer biomarkers like the sialyl Lewis^a^ epitope (Neu5Acα2-3Galβ1-3(Fucα1-4) GlcNAc), the sialyl Lewis^x^ motif, a structural isomer of Lewis^a^, and the sialyl-Tn epitope (Neu5Acα2-6GalNAc) have been reported to be used as targets for cancer immunotherapy in preclinical, as well as in clinical vaccine evaluation [26,27,28,29]. 

### 1.2. Siglecs

Sialic acid-binding immunoglobulin-like lectins (Siglecs) are a group of cell surface proteins that can bind to sialic acids. The term Siglec was proposed by Crocker, Varki, and other researchers in the glycobiology field after a high sequence similarity between the sialic acid-binding proteins sialoadhesin, CD22, CD33, and the myelin-associated glycoprotein (MAG) was found. These proteins were consequently named Siglec-1-4 [30]. All Siglecs are type-1 single-pass transmembrane proteins that consist of a variable (V-set) domain and a various number (1–16) of C2-set immunoglobulin-like (Ig) domains. A conserved arginine residue in the V-set domain is essential for optimal sialic acid-binding [31,32]. The Siglec family can be divided into two subgroups. Siglec-1, -2, -4 and -15 are highly conserved among different species [33]. In contrast, the CD33(Siglec-3)-related Siglecs that are found on chromosome 19 on a gene cluster are more rapidly evolving [34]. It is worth noting that mice have a different set of Siglecs than humans, and many Siglecs do not have true homologs between humans and mice. Moreover, unlike humans, mice can synthesize Neu5Gc in addition to Neu5Ac. Therefore, Siglec functions studied in mouse models can often not be adapted to humans [35]. In humans and primates, 17 Siglec genes have been discovered so far. Siglec-13 and Siglec-17 are pseudogenes, and the corresponding proteins are not present in humans [36]. Siglec-12 is only expressed in some humans and has a mutation in the binding domain that makes it unable to bind to sialic acids [37]. So overall, 14 functional human Siglecs exist that have different binding specificity for several sialylated glycans [38]. Most Siglecs have the function to mediate inhibitory signals, while some act as an activating receptor and others are involved in cell-cell adhesion [39,40]. Inhibitory Siglecs have a membrane-proximal immunoreceptor tyrosine-based inhibitory motif (ITIM, I/L/S/VXYXXL/V, where X can be any amino acid) and a membrane-distal ITIM-like motif in their cytoplasmic domain. Upon ligand-binding, the tyrosine residue of the ITIM is phosphorylated and recruits different phosphatases [41].

Siglecs bind sialic acid molecules on other cells (trans-interaction), but can also bind residues on the same cell (cis-interaction). These cis interactions might fulfill a masking function on the surface of the cell resulting in a potential inhibition of sialic acid ligand binding [42]. Treatment with a neuraminidase, an enzyme hydrolyzing the covalent bond between the sialic acid and the glycoconjugate, removes these cis ligands. Moreover, it has been shown for Siglec-2 on B cells that cellular activation can lead to demasking in vivo [43,44]. Sialyltransferase expression can regulate the masking of Siglecs as it could be shown that knockout of a certain sialyltransferase in mice constitutively unmasked Siglec-2 on B cells [45]. Collins et al. could further show that high-affinity Siglec-ligands are able to bind to Siglecs even when they are masked [46]. Siglecs are mainly expressed on cells of the haematopoietic and immune system, and most human immune cells express at least one Siglec [40]. As sialic acids are present in all human cells, Siglec-binding can act as a recognition mechanism for the immune system to identify healthy endogenous cells.

### 1.3. NK Cells

Natural Killer (NK) cells are cells of the innate immune system. With their ability to kill virus-infected cells and tumor cells, NK cells play a major role in defense against viral infection and tumor progression. NK cells are derived from common lymphoid progenitor cells and mature in the bone marrow and secondary lymphoid organs. During different development stages, progenitor cells become immature NK cells, which give rise to mature NK cells. Expression of CD56 marks the final formation of mature NK cells [47]. CD56 is identical to the neural cell adhesion molecule (NCAM) that has been well studied in the central nervous system [48]. Based on CD56 expression, human NK cells in the blood are generally divided into two subpopulations. About 10% of the blood NK cells have a high CD56 expression (CD56^bright^), and 90% of the NK cells express lower amounts of CD56 (CD56^dim^). CD56^bright^ NK cells produce high levels of different cytokines to modulate the function of other immune cells, whereas CD56^dim^ NK cells have a higher cytotoxic potential [49]. NK cells do not recognize specific antigens, but have different activating and inhibitory receptors that can detect several ligands on potential target cells. Activating receptors are coupled with adapter molecules for intracellular signaling that can activate different signaling pathways leading to NK cell activation. Inhibitory receptors have an ITIM in their cytoplasmic domain that can recruit phosphatases, inhibiting activating signals. [50,51]. The balance between activating and inhibitory signals determines, whether the NK cell is activated and a potential target cell is killed. While activating receptors detect ligands present on the surface of transformed, virus-infected, or stressed cells, inhibitory receptors recognize ligands that are present on almost all healthy cells, for example, the human leukocyte antigen (HLA) molecules [52]. NK cells require cell-cell contact to kill target cells. At first, an immunological synapse is formed between the two cells. If the NK cell is activated, different adhesion molecules cluster rapidly at the synapse leading to firm adhesion. After the reorganization of actin, lytic granules are brought to the immunologic synapse, and their content is released leading to the degranulation of the NK cell [53]. These lytic granules are secretory lysosomes containing perforin, granzymes, and other molecules that promote the death of the target cell [54]. If the NK cell detects a healthy cell, the inhibitory signals predominate. As a result, the transport of lytic granules is inhibited, the immunologic synapse is destabilized, and adhesion is reduced [55,56]. Subsequently, both cells detach without lysis of the target cell. To avoid the existence of auto-reactive NK cells, only NK cells that express inhibitory receptors are becoming fully functional during development. This process is termed NK cell education or licensing [57]. Besides, NK cells can kill target cells coated with antibodies via antibody-dependent cellular cytotoxicity (ADCC) [58]. To fulfill their role in the immune system, NK cells not only kill transformed or infected cells, but also produce different cytokines, like tumor necrosis factor-α, interferon-γ (IFN-γ), interleukin-10 (IL-10), and granulocyte-macrophage colony-stimulating factor (GM-CSF) to modulate the function of other immune cells [59]. Moreover, the activity of NK cells is also regulated by various cytokines, such as IL-2, IL-12, IL-15, IL-18 IFN-α, and IFN-β [60]. IL-12, IL-18, and IFN-α, for example, are produced by dendritic cells showing that these have an influence on NK cells [61,62,63]. Besides, NK cells impact the maturing process of dendritic cells via soluble factors, such as TNF and INF-γ, but also via cell-cell-contact. Another aspect of DC-NK-cell interaction is that immature DCs are prone to NK-cell-mediated cytolysis, whereas mature DCs are protected, due to their different HLA class I expression levels [64,65,66]. 

Human NK cells express receptors that can bind sialic acids, but also have sialylated molecules on their surface, which both contribute significantly to NK cell function. Therefore, sialic acid-binding receptors and sialic acids on NK cells will be separately described in the following sections.

## 2. Sialic Acid-Binding Receptors on NK Cells

### 2.1. Siglec-7

Siglec-7 (also termed CD328 or p75/AIRM1) is a type-1 membrane protein that is expressed on a vast majority of NK cells and a small number of CD8^+^ T cells and is also present at lower levels on granulocytes and monocytes. The protein of 467 amino acids is an inhibitory receptor consisting of two C2-set domains and one N-terminal V-set domain. Furthermore, Siglec-7 has an ITIM and an ITIM-like motif in the cytoplasmic domain [67,68]. Even though the percentage of Siglec-7^+^ NK cells is the same in the two NK cell subsets, CD56^dim^ NK cells have a higher density of Siglec-7 on their surface compared with CD56^bright^ NK cells, which can be observed by higher median fluorescence intensity [69,70]. Expression of Siglec-7 on NK cells is regulated at the transcriptional level, and DNA methylation at the promoter region results in a reduced Siglec-7 amount on the cell surface [71]. The binding domain of Siglec-7 is well characterized, and different crystal structures are available that can help to understand the binding specificity and to identify possible substrates and inhibitors [72,73]. In addition to the sialic acid-binding site around the essential arginine residue (R124), Siglec-7 has another binding site in the V-set domain, and both binding sites contribute to glycan recognition [74]. The signaling of Siglec-7 is similar to other inhibitory Siglecs, and is mediated through the membrane-proximal ITIM. When ligand-binding occurs, the tyrosine residue of the ITIM is phosphorylated and recruits the tyrosine phosphatases SHP-1 and SHP-2, which are able to antagonize the signals induced by activating receptors [75].

Siglec-7 preferentially binds to α-2,8-disialyl and branched α-2,6-sialyl residues [76]. Different ligands for Siglec-7 have been identified, among them the sialoganglisoside GD3, GD2, GT1b, DSGb5, and DSL_c_4, and the carbohydrate structures disialyl Lewis^a^ and disialyl Lewis^c^ [73,77,78]. Experiments with recombinant Siglec-7 showed that ligands for Siglec-7 are highly expressed on several tumor cell lines, including leukemic and melanoma cell lines. Moreover, Siglec-7 ligands are found on primary tumor cells derived from patients with acute myeloid leukemia (AML), chronic lymphocytic leukemia (CLL), or malignant melanoma [67,68]. Signaling by Siglec-7 is one of the immune checkpoints that can be targeted to enhance the anti-tumor activity of NK cells [79]. Due to the fact that Siglec-7 is an inhibitory receptor on NK cells, the expression of Siglec-7 ligands on tumor cells can protect them from NK cell-mediated lysis. If these ligands are cleaved by neuraminidase treatment, no Siglec-7 binding and signaling can occur, and consequently, killing by NK cells is highly improved. Blocking of Siglec-7 binding by antibodies also improves killing efficiency against K562 and HeLa cells that express high levels of Siglec-7 ligands [80]. In addition, Hudak et al. could show that increasing sialylation of tumor cells by the incorporation of synthetic glycopolymers could protect these cells from NK cell killing via engagement with Siglec-7 [81]. 

Besides the interaction with ligands on tumor cells, it has been observed that Siglec-7 can also recognize sialic acid structures on different bacteria, including *Campylobacter jejuni* and *Pseudomonas aeruginosa*. Because of Siglec-7, NK cells are able to bind to these bacteria, but the consequence of this binding is not fully understood, yet [82,83]. Fong et al. described the interaction of Siglec-7 on NK cells with a protein from the human pathogen group B *Streptococcus*. In this case, binding of Siglec-7 is assumed to reduce the formation of the inflammasome complex and inhibit pyroptosis of NK cells. Therefore, it seems that the expression of Siglec-7 ligands is a mechanism by which the pathogen can suppress an immune response [84]. Because tumor cells and pathogens can utilize Siglec-7 to escape the immune system, blocking of Siglec-7 binding might be a possible tool for cancer treatment. Therefore, high-affinity Siglec-7 ligands have been designed that are able to overcome the Siglec-7-dependent inhibition of NK cells [85,86]. 

Apart from its functional role, Siglec-7 can be used as a marker to identify functional NK cells. Siglec-7^+^ NK cells express more activating receptors like NKp30 and NKp46 and have a stronger ability for degranulation. Moreover, they produce more IFN-γ than Siglec-7^−^ NK cells [69]. In HIV-1-infected viremic patients, the number of Siglec-7^+^ NK cells is markedly decreased, and low amounts of Siglec-7 can be used, together with a reduced CD56 expression, as a marker for a dysfunctional NK cell subset [87]. In line with this, HIV elite controllers, untreated infected individuals with undetectable HIV viremia, have a higher number of Siglec-7^+^ NK cells. As this cell population is generally more active, it might contribute to the control of HIV replication [88]. It is known that HLA and non-HLA molecules can be involved in NK cell education [57]. Siglec-7 is expressed on a vast majority of NK cells, and sialic acids are widely expressed in healthy cells. Given the fact that NK cells that do not express Siglec-7 are less functional, it would be interesting to analyze if Siglec-7 could play a role in HLA-independent NK cell education. Siglec-7 is also reduced in patients with primary hepatocellular carcinoma (HCC), where the frequency of Siglec-7^+^ NK cells in the peripheral blood is much lower compared to healthy donors. Siglec-7^+^ NK cells show a more activated phenotype. Therefore, a reduced Siglec-7 expression in HCC could predict NK cell dysfunction, also supporting the utilization of Siglec-7 as a marker for functional NK cells [89]. Patients with a chronic hepatitis C virus infection also have a reduced expression of Siglec-7 on NK cells, which correlates with markers of liver inflammation and fibrosis [90]. Besides, in obesity, which is associated with a reduced NK cell function, Siglec-7 expression on CD56^bright^ NK cells is reduced [70]. Siglec-7 is also downregulated during infection with the human cytomegalovirus (HCMV), and in HCMV-infected patients, an expansion of Siglec-7^−^ NKG2C^+^ NK cells is observed [91]. In contrast to the Siglec-7^-^ NK cells described above, this subset displays an enhanced function with a more efficient killing via ADCC and an elevated IFN-γ production. These cells that help to control HCMV infection are called “memory-like” NK cells as they show some characteristics of adaptive immunity (persistence over time, clonal expansion, enhanced function, and epigenetic modifications) [92].

### 2.2. Siglec-9

Siglec-9 (CD329) shares over 84% of its protein sequence with Siglec-7 and also contains two C2-set domains and one N-terminal V-set domain, as well as an ITIM and ITIM-like motif. The protein consists of 463 amino acids and has an equal binding preference for α2,3- and α2,6-linked sialic acids [93]. Siglec-9 is highly expressed on monocytes in which more than 90% of the cells belong to the Siglec-9^+^ population. Furthermore, Siglec-9 is also expressed on NK cells, but the expression seems to be restricted to the CD56^dim^ subset, while CD56^bright^ NK cells have no or only a weak Siglec-9 expression [94]. Similar to Siglec-7, ligands for Siglec-9 are present on different tumor cell lines and primary tumor cells, particularly on cells derived from hematologic malignancies or melanoma [80,95]. Based on glycan arrays, binding of Siglec-9 to different sialogangliosides like GD1a and GT1b, as well as to sialyl Lewis^x^ structures, have been observed [96]. Moreover, Siglec-9 is able to bind mucins, a family of heavily glycosylated proteins, on cancer cells. The mucin MUC-16, which is highly expressed in ovarian cancer, is recognized by Siglec-9 on NK cells and reduces the NK cell-mediated killing of the tumor cells [94,97]. Blocking of Siglec-9 interaction by specific antibodies enhances the lysis of sialylated tumor cell lines by NK cells [80]. Furthermore, inhibitory effects after binding of Siglec-9 to its ligands have been reported in T cells and neutrophils [98,99]. CD56^dim^ Siglec-9^+^ NK cells exhibit a more mature phenotype and seem to have a higher chemotactic capacity. The number of Siglec-9 expressing NK cells in the blood is lower in patients with colon adenocarcinoma or malignant melanoma even though the overall number of NK cells is comparable to healthy donors [80]. In patients with chronic Hepatitis B virus infection (CHB), the number of Siglec-9^+^ NK cells is reduced and correlates negatively with virus replication status [100].

Due to high heterogeneity of different donors, NK cell lines like NK-92 are sometimes used to study NK cell function. Interestingly, the human NK cell lines NK-92 and NKL do not express Siglec-7 or Siglec-9 [70], and require transfection to be used for the study of Siglec function [81]. Nevertheless, Siglec-7 can be found on the IL-2-independent cell line NK-92 MI after a long in vitro culture time [101]. Even though Siglec-7 and Siglec-9 have high similarities in gene and protein sequence, their expression pattern, binding specificity, and role on NK cell function differ. Compared to Siglec-7, which is extensively studied in the case of structure and function, the role of Siglec-9 on NK cell function is only partly understood (reviewed by Zheng et al. [102]). Moreover, no crystal structure of Siglec-9 is available. 

**Figure 2:**
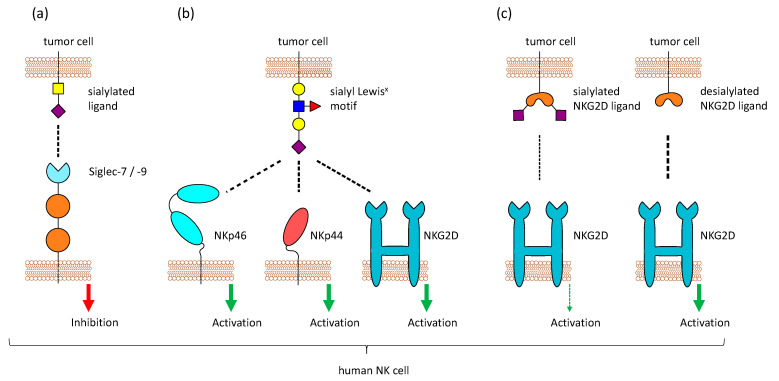
Sialic acid-binding receptors on Natural Killer (NK) cells. Different sialic acid-binding receptors are present on NK cells. (**a**) NK cells express Siglecs (mostly Siglec-7 and Siglec-9), which bind sialic acids and mediate inhibitory signals. Sialylated tumor cells can exploit these receptors to escape NK cell-mediated killing (**b**) One particular sialic acid motif, sialyl Lewis^x^, is detected by the activating receptors NKp46, NKp44, and NKG2D, leading to NK cell activation. (**c**) NKG2D usually binds stress-induced ligands like MICA, MICB, and ULPs. Binding of NKG2D to its ligands on tumor cells is reduced when these ligands are sialylated.

The inhibitory receptors Siglec-7 and Siglec-9 on NK cells can bind to sialic acid structure and help to recognize healthy cells, but sialylated tumor cells can exploit this mechanism to be protected against NK cell-mediated killing (Figure 2a). Therefore, the hypersialylation of cancerous cells and their interaction with Siglecs on NK cells might be a good target for cancer treatment. This could be achieved by enzymatic desialylation of tumor cells, blocking of Siglec-binding, or inhibition of sialyltransferase activity (reviewed by Daly et al. [103]). Because ligands for Siglec-7 and Siglec-9 are highly expressed on different tumor entities, Siglec-7/9-based chimera constructs have been tested for the use in chimeric antigen receptor (CAR)-T cell therapy in a recent study. The modified T cells could show promising anti-tumor activity in vitro and in vivo and might be a tool to target highly sialylated tumors [95]. 

### 2.3. Other Siglecs on NK Cells

Most studies of Siglecs on NK cells are restricted to Siglec-7 and Siglec-9, but an expression of other Siglecs in NK cells has also been described. Siglec-3 (CD33), an inhibitory receptor with one C2-set domain and one V-set domain [42], is expressed at low levels in early NK cell development, but is nearly absent in the CD56^bright^ and CD56^dim^ NK cell subsets in the peripheral blood [104]. The Siglec-3 expression in NK cells during different development stages also varies between different tissues. For example, Siglec-3 is expressed significantly longer on NK cells in hepatic lymph nodes than in other tissues [105]. Furthermore, CD33 is expressed by NK cells derived from the peripheral blood, when they were stimulated in vivo [106,107]. The function of Siglec-3 on NK cells is not extensively investigated, but studies with the human NKL cell line could reveal a possible role of Siglec-3 in inhibiting signals from the activating receptor NKG2D [108].

Another Siglec that was reported to be present on NK cell is Siglec-10, which consists of four C2-set and one V-set domain and binds α2,3- and α2,6-linked sialic acids. It is an inhibitory receptor expressed on eosinophils, monocytes, and B-cells and was also reported to be highly present on a small subset of CD56^−^ and CD16^+^ lymphocytes [109]. Siglec-10 could also be found on NK cells of patients with hepatocellular carcinoma. The protein was present on about 40% of the NK cells in the tumor tissue, and to a lesser extent, in the surrounding tissue. Furthermore, Siglec-10^+^ NK cells were less functional, and a high Siglec-10 expression was a negative prognostic factor for survival after tumor resection [110].

As previously mentioned, Siglec-17 is a pseudogene in humans because of an interrupted open reading frame. Nevertheless, the mRNA was still expressed exclusively on NK cells, suggesting that the corresponding protein might have been present on NK cells of hominin ancestors [36].

In NK cells, Siglec-7 and Siglec-9 are predominantly studied because most experiments are performed with NK cells derived from the peripheral blood. Nevertheless, other Siglecs are also present on NK cells in different tissue, and their role on NK cell function should also be considered. All Siglecs that were found to be expressed on NK cells are summarized in Table 1.

### 2.4. Other NK Cell Receptors that Bind Sialic Acids 

Aside from Siglecs, other NK cell receptors have been reported to bind to sialic acids, for example, CD94 and NKG2D. CD94, which is expressed on the majority of NK cells, forms heterodimers with members of the NK group 2 (NKG2) family and these dimers usually bind to the non-classical HLA-E molecule. NKG2A and B have an ITIM and mediate inhibitory signals, while NKG2C, E, and H function as activating receptors [114]. In contrast, the activating receptor NKG2D does not form a complex with CD94 and is present as a homodimer. It binds to MHC class-I-chain related proteins A and B (MICA, MICB), retinoic acid early transcript 1 (Rae1), and some UL16-binding proteins (ULBPs) that are upregulated by transformed or stressed cells [115]. Both CD94 and NKG2D were also reported to bind sialic acids, in particular the sialyl Lewis^x^ motif on multi-antennary *N*–glycans [116,117]. Moreover, binding of the activating receptors NKp44 and NKp46, but not NKp30, to the same sialic acid structures was also observed [118,119] (Figure 2b). The overexpression of sialyl Lewis^x^ on tumor cells made them more susceptible to NK cell-mediated lysis [120]. In addition, studies in mice could also reveal that tumor cells transfected with a glycosyltransferase to express a high level of sialyl Lewis^x^ are lysed more efficiently by NK cells, because of CD94 recognition [121]. In contrast to that, NKG2D binding is highly increased after desialylation of tumor cells [122] (Figure 2c). Removal of sialic acids on tumor cells reduces the binding of Siglec-7 and Siglec-9 and simultaneously enhanced the binding of NKG2D, leading to better lysis by NK cells [123]. Even though this seems to be contrary to the binding of NKG2D to sialyl Lewis^x^, it could be possible that NKG2D can recognize both the expression of stress-induced ligands like MICA and the abnormal high expression of sialyl Lewis^x^. A high level of sialylation changes the glycocalyx of a cell and inhibits the interaction of NKG2D with the stress-induced ligands, while providing glycans that can be recognized by Siglecs. This can protect tumor cells from the detection and lysis by NK cells. Approaches have been made to remove sialic acids specifically from tumor cells by fusing a neuraminidase to an antibody against human epidermal growth factor receptor 2, which is highly expressed in breast cancer. This construct was able to selectively desialylate tumor cells and make them prone to NK cell killing [123].

As described in this section sialylation of target cells and their recognition by sialic acid-binding proteins on NK cells have an effect on the lysis of the target cells. Although studies in this field mainly focus on interactions of these sialic acid-binding proteins and sialic acids on potential target cells, it could also be possible that they play a role in the interaction with other immune cells. For example, it is known that dendritic cells, which are highly sialylated, and NK cells have reciprocally regulatory effects through cytokines, but also through cell-cell interaction on each other [64,124]. However, the exact interactions between sialic acid-binding proteins on NK cells and sialylated residues on dendritic cells still needs to be further investigated. 

## 3. Sialic Acid on NK Cells

### 3.1. Different Sialylation of NK Cell Subsets and Changes during NK Cell Activation

NK cells are sialylated, and lectin staining suggests that the two NK cell subpopulations have a different level of sialylation—more precisely, CD56^bright^ NK cells have more sialic acids on their surface than CD56^dim^ NK cells [70]. As previously described, the transfer of CMP-sialic acid to different glycan structures is catalyzed by sialyltransferases in the Golgi apparatus. After activation of NK-92 cells with IL-2, three sialyltransferases were found to be downregulated, in particular ST8Sia1, ST6Gal1 and ST3Gal1. Moreover, the sialylation is changed by activation, as the amount of α2,6-linked sialic acids is increased, whereas the amount of α2,3-linked sialic acids is not changed [125]. Different expression of sialyltransferases can change the sialylation of particular molecules. Altered sialylation can then lead to changes in protein stability and function. 2B4 is one NK cell receptor whose binding affinity can be impacted by sialylation. It can bind to CD48, a glycosyl-phosphatidylinositol-anchored molecule, and is able to activate NK cells. An N-linked glycosylation on 2B4 is essential for this interaction. Interestingly, when sialic acid residues are removed from 2B4, binding to CD48 is highly increased [126]. Sialylation can also change the half-life of certain proteins. For example, when the intercellular adhesion molecule 1 (ICAM-1) is sialylated by ST6Gal1, its stability is increased, leading to a higher abundance [127].

### 3.2. Masking of Siglecs

**Figure 3:**
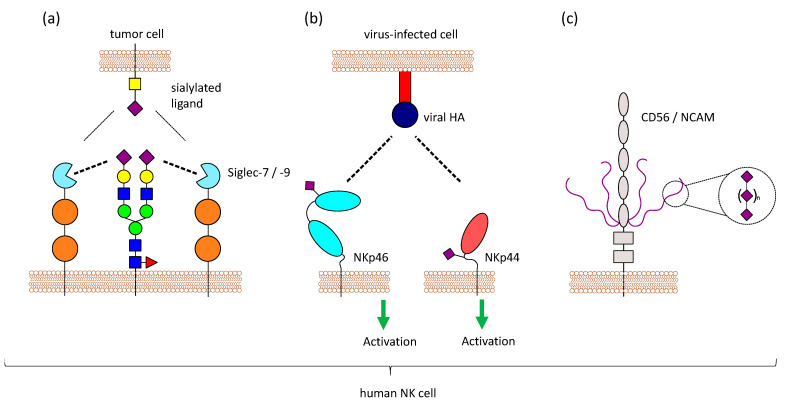
Sialic acids on NK cells. NK cells have sialic acids on their surface, which can influence NK cell functions in different ways. (**a**) Siglecs can not only bind to molecules on other cells (trans), but also to molecules on the same cell (cis). Masking can regulate Siglec function, since trans-ligands have to compete with the masking ligands present on NK cells. (**b**) NKp46 and NKp44 are sialylated, and it has been shown that these activating receptors interact with viral proteins like HA in a sialic acid-dependent manner. Virus-infected cells producing viral HA can be detected by NK cells via NKp44 and NKp46. (**c**) Furthermore, NK cells express CD56/NCAM, which carries a special form of glycosylation called polySia (long chains of sialic acid molecules attached to via α-2,8 linkage). PolySia on NCAM has been well studied in the central nervous system, where it is involved in regulating cell adhesion, but the function of this modification on NK cells is still unknown.

Another functional role of sialic acids on NK cells could be the masking of Siglecs, as previously mentioned. The predominantly expressed Siglecs on NK cells, Siglec-7 and Siglec-9, both are masked by yet unknown ligands [93,128]. Based on the binding specificity of Siglec-7 and mass spectrometry analysis, Avril et al. suggest a 2,8-disialic acid structure as the masking ligand [129], but it is still unclear, whether one or multiple ligands are involved in masking of Siglec-7 and Siglec-9. Treatment of NK cells with neuraminidase results in a demasking of Siglec-7 which is then able to bind to ligands on other cells. This has been observed with the sialoganglisosides GD3 and DSGb5 on tumor cells, where a strong inhibitory effect on NK cell-mediated lysis was only visible after demasking of Siglec-7 [128,130]. In contrast to that, it has been reported that Siglec-7 can bind to different ligands, even when masked, without prior enzymatic treatment [80,81]. Various ligands for Siglec-7 and Siglec-9 exist on potential target cells, which have to compete with the masking ligands present on NK cells (Figure 3a). Therefore, the requirement for demasking of Siglec-7 and Siglec-9 might depend on the binding affinity of the cis and trans ligands. It is worth noting that GD3, one ligand of Siglec-7, is also present on NK cells as 7-O-acetyl-GD3 [131,132], suggesting a possible role of GD3 in masking Siglec-7. ST8Sia1, the sialyltransferases responsible for the synthesis of GD3, and the ganglioside itself are markedly decreased when NK cells are activated with IL-2 [125]. 

### 3.3. Interaction of Viral Proteins with Sialylated Receptors on NK Cells

Natural cytotoxic receptors (NCRs) belong to the activating NK cell receptors and are type-1 membrane proteins. The group of NCRs consists of the receptors NKp30, NKp44, and NKp46, which can recognize several ligands on tumor cells and virus-infected cells. While NKp30 and NKp46 are constitutively expressed by NK cells, NKp44 is upregulated during NK cell stimulation. NCRs are coupled with adapter proteins like CD3ζ and the FC receptor common γ (NKp30, NKp46) or DAP12 (NKp44) for downstream signal transduction [133].

NKp46 is sialylated and recognizes haemagglutinin (HA) of influenza virus and haemagglutinin neuraminidase (HN) of the parainfluenza virus. This interaction depends on the sialylation of NKp46, and neuraminidase treatment of NKp46 reduces the binding [134]. Sialylation on a specific O-glycosylation site (Thr225) of NKp46 is important for the interaction with viral HA. Even though Thr225 is also involved in the recognition of tumor ligands, the binding to tumor cells does not depend on sialylation of NKp46 [135]. Binding of NKp46 to HA strongly depends on the sialic acid linkage, as the recombinant expression of NKp46 in different cells leads to different glycan structures and results in different binding of NKp46 to HA [136]. NKp46 was further shown to recognize cells infected with reovirus by binding to reovirus sigma1 protein. Sialylation on Thr225 was also crucial for this interaction [137]. NKp44 was also shown to interact with viral HA and HN in a sialic acid-dependent manner. However, NKp30, the other member of the NCR family, is also heavily glycosylated, but does not show binding to HA or HN [138]. Virus-infected cells that produce viral HA can be detected by NK cells via NKp44 and NKp46, leading to the lysis of the infected cells [134,138] (Figure 3b). Influenza HN which also interacts with NKp44 and NKp46, has an opposite effect. The neuraminidase activity leads to desialylation of NKp44 and NKp46, which results in reduced recognition of viral HA and impairs the killing of virus-infected cells by NK cells [139,140]. In contrast to that, NKp46 and NKp44 can bind to HN of the Newcastle disease virus, and this interaction increases the susceptibility of virus-infected tumor cells to NK cell-meditate lysis [141]. 

Proteins that bind sialic acids on glycoproteins and gangliosides are found on many different viruses and used for entry into the host cell [142]. As these viral proteins are also expressed in cells during viral infection, the interaction of NKp44 and NKp46 on NK with these proteins might represent a way how virus-infected cells can be detected and killed by NK cells. Nevertheless, in the case of influenza, viral HA can have a dual role. It can be recognized by NKp44 and NKp46 leading to enhanced killing of virus-infected cells, but viral HA protein alone or attached to virions can also inhibit the cytotoxic activity of NK cell. This effect is mediated through the degradation of the CD3ζ chain via the lysosomal pathway [143]. Consequently, free influenza virions and free HA can also act negatively on NK cell function. 

Other NK cell receptors that have been shown to interact with influenza HA, and therefore, can recognize virus-infected cells, are 2B4 (CD244) and NTB-A. Similar to NKp44 and NKp46, the interaction depends on the sialylation of 2B4 and NTB-A, and desialylation by NA reduces the binding [144]. 

### 3.4. CD56/NCAM is Polysialylated

NK cells in the peripheral blood are generally defined by the presence of CD56/NCAM and the absence of CD3. There are three different isoforms of NCAM with a molecular mass of 120, 140, and 180 kDa. The 140 kDa isoform is expressed on NK cells [145]. As previously mentioned, CD56/NCAM carries a special posttranslational modification, polysialic acid (polySia), with long chains of sialic acid molecules attached to each other via α-2,8 linkage, and this modification is also found on CD56/NCAM of human NK cells [146]. The extracellular part of CD56/NCAM consists of 5 immunoglobulin-like domains (Ig1–5) and 2 fibronectin-type III repeats (FN1, FN2). PolySia on CD56/NCAM is only found on 2 *N*-glycosylation sites that are both located in the Ig5 domain [147] (Figure 3c).

Apart from being a marker for NK cells and their subpopulations, the function of CD56/NCAM on NK cells is still to be investigated. CD56/NCAM can bind to other CD56/NCAM molecules, and these homophilic interactions were also reported in NK cells, which can bind to CD56/NCAM positive tumor cells. The impact of this binding seems to be controversial, since some studies show that CD56 expression on tumor cells makes them more sensitive for NK cell-mediated lysis [148,149], while experimental overexpression of CD56/NCAM can be protective for some tumor cell lines [150]. Besides, a recent publication could show that CRISPR-Cas9-mediated deletion of CD56/NCAM in the NK cell line NK-92 leads to a reduced killing of CD56 negative tumor cells. In contrast, loss of CD56/NCAM on primary NK cells had no impact on cytotoxic activity [151]. With experiments on NK cell lines and primary cells, Mace and colleagues could show that CD56/NCAM is involved in migration during NK cell development [152]. Moreover, because CD56/NCAM on NK cells seems to bind to fungal mold *Aspergillus fumigatus*, a role as a pattern recognition receptor is also discussed [153]. Additionally, a role for CD56/NCAM in cell signaling was assumed, since phosphorylation of Pyk2, a tyrosine kinase was decreased in CD56/NCAM knockout NK cells and could be rescued by CD56/NCAM transduction [151].

This shows that the functional role of CD56/NCAM on NK cells is not fully understood, and furthermore, it has to be taken into consideration that this molecule is polysialylated and that the degree of sialylation might also have an impact on protein function. Modification of CD56/NCAM with polySia results in a negative charge and has been shown to reduce cell adhesion to extracellular matrix proteins in neuroblastoma cell lines [154]. Moreover, polysialylation of CD56/NCAM also inhibits the homophilic interactions leading to reduced cell-cell contacts [155,156]. PolySia might also regulate the turnover of NCAM as it has been observed that polysialylated NCAM has a longer half-life [157]. Two sialyltransferases, ST8Sia II and ST8Sia IV, can synthesize polySia on CD56/NCAM, whereas only ST8Sia IV is expressed in human NK cells [125,158]. When NK cells are activated with IL-2, the expression of polySia and CD56/NCAM is increased. Moreover, the degree of polymerization, the length of the sialic acid chains, is changed [146]. As IL-2 activation does not have an impact on the expression of ST8Sia IV, the higher polySia amount is probably due to the higher expression of the carrier molecule CD56/NCAM [125]. The functional reason for the increased expression during activation remains unclear, but it could probably be that it contributes to a higher migration capacity because of a reduced adhesion.

As human NK cells express Siglec-7, which is known to bind α-2,8-linked sialic acids, it has been hypothesized that polySia on CD56/NCAM might act as a masking ligand for Siglec-7. Neuraminidase treatment, which cleaves all sialic acids, reduces the killing of NK cells against K562 tumor cells by unmasking Siglec-7. In contrast, specific removal of polySia had no impact on the killing efficiency. Therefore, polySia seems not to be the ligand masking Siglec-7 [158]. However, another functional role of polySia on NK cell-mediated killing cannot be excluded. 

The function of CD56/NCAM on NK cells is only partly understood, and it is important to also investigate the role of polySia as this modification has been shown on other cells to regulate cell-matrix and cell-cell interactions, as well as the turnover of NCAM. 

## 4. Conclusions

Sialic acids can be involved in NK cell function in two different ways. NK cells express sialic-acid-binding receptors and also have sialic acids on their surface themselves. 

Two well-studied receptors that can bind sialic acids are Siglec-7 and Siglec-9, which have an inhibitory function on NK cells. Tumor cells that are often highly sialylated can escape NK cell-mediated lyses by expressing high amounts of Siglec ligands. Additionally, high levels of sialic acids can be beneficial for tumor cells, since binding of the activating receptors NKG2D to its ligands on tumor cells is reduced when they are sialylated. Nevertheless, sialyl-Lewis^x^, a particular sialylated structure, is detected by activating receptors resulting in enhanced killing by NK cells. Like all human cells, NK cells have sialylated molecules on their surface. The sialylation of NK cells is different between CD56^dim^ and CD56^bright^ NK cells and is changed during NK cell activation. Sialic acids can regulate receptor activity and abundance. Moreover, some activating receptors like NKp44 and NKp46 require sialylation to mediate binding to viral HA and detect virus-infected cells. Besides, human NK cells express CD56/NCAM, which carries the polySia modification. While polySia is known to regulate cell adhesion in cells of the central nervous system, the function of polySia on NK cells remains unknown.

The contribution of sialic acids to NK cell functions is complex and diverse. Understanding the role of sialic acids on NK cells could help to enhance NK cell activity, for example, through the selective desialylation of tumor cells or the blockade of Siglec-binding. Therefore, the impact of sialic acids on the interaction between NK cells and potential target cells needs to be further investigated. In particular, the identification of masking ligands for Siglec-7 and Siglec-9 and their regulation remains one of the main topics for further research. Another important question that still needs to be discovered is the functional role of polySia on NK cells. 

## Figures and Tables

**Table 1:** Siglecs on NK cells.

	Structure	Identified Ligands (Examples)	Expression on NK Cells	Crystal Structures ^1^	Ref.
**Siglec-3** **(CD33)**	1 V-set 1 C2-set	sialyl-Tn, complement component 1q	early NK cells, absent on NK cells in the blood	6D48, 5IHB, 5J06, 5J0B, 6D4A, 6D49	[42,104,111,112]
**Siglec-7** **(CD328)**	1 V-set 2 C2-set	GD3, GD2, GT1b, DSGb5, DSL_c_4, disialyl Lewis^a^, disialyl Lewis^c^	almost all NK cells, higher expression on CD56^dim^ NK cells	1O7V, 1O7S, 2G5R, 2DF3, 2HRL, 1NKO	[67,69,70,73,77,78]
**Siglec-9** **(CD329)**	1 V-set 2 C2-set	GT1b, GD1a, sialyl Lewis^x^, MUC16	CD56^dim^ NK cells, low or no expression on CD56^bright^ NK cells	n.a.	[93,94,96,97]
**Siglec-10**	1 V-set 4 C2-set	CD24, GT1b	NK cells in tumor tissue, absent on NK cells in the blood	n.a.	[109,110,113]

^1^ Files from the Protein Data Bank.
